# De novo assembly of transcriptome during regeneration post-arm amputation in the starfish, *Asterias amurensis*

**DOI:** 10.1186/s12863-025-01340-3

**Published:** 2025-07-08

**Authors:** Mi Jeong Jo, Hye-Jin Go, Jeong Gyu Kim, Gun-Do Kim

**Affiliations:** 1 Department of Microbiology, Pukyong National University, Busan, 48513 South Korea; 2 Industry-University Cooperation Foundation, Pukyong National University, Busan, 48513 South Korea; 3 Institute of Marine Living Modified Organisms, Pukyong National University, Busan, 48513 South Korea

**Keywords:** *Asterias amurensis*, Starfish, De novo assembly, Transcriptomic analysis, Regeneration

## Abstract

**Objectives:**

This study investigates the nerve cord transcriptome of *Asterias amurensis* to explore its regenerative abilities. By comparing gene expression between a normal group and a group 72 h post-amputation, key genes involved in regeneration were identified. Functional annotation using GO, KEGG, NR, and UniProt databases provided insights into the biological roles of these genes. This research enhances the understanding of *A. amurensis* regeneration and highlights the need for further transcriptome analysis across different tissues.

**Data description:**

*A. amurensis*, a starfish species found in the northwestern Pacific, is known for its strong predatory behavior and impact on marine biodiversity. In this study, individuals were divided into a normal group and a 72-hour post-amputation group. De novo transcriptome assembly of the nerve cord identified 257,769 unigenes, which were functionally annotated using GO, KEGG, NR, and UniProt databases. Since only nerve cord tissue was analyzed, additional transcriptome studies on various tissues are required for a more comprehensive understanding of *A. amurensis* biology.

## Objective

Starfish (*Asterias amurensis*), belonging to the phylum Echinodermata, play a crucial role in marine ecosystems by regulating shellfish populations [1]. They possess a unique water vascular system that facilitates movement and predation [2]. Notably, their remarkable regenerative ability allows them to regrow lost arms or even parts of the central disk, making them a valuable model for studying cell proliferation, differentiation, and tissue reorganization [3]. Research on *A. amurensis*, a highly reproductive species found in the northwestern Pacific, is particularly important due to its strong predatory behavior and impact on biodiversity [4]. With advances in next-generation sequencing (NGS), transcriptomic studies have provided deeper insights into biological processes, such as nervous system function, regeneration, and stress responses [3, 5]. Previous studies, including transcriptome decoding of *Asterias rubens*, have laid the foundation for understanding gene functions and evolution in echinoderms [6]. This study aims to analyze the transcriptome of the nerve cord in *A. amurensis*, focusing on differentially expressed genes (DEGs) during the regeneration process. By identifying key genetic components, this research will contribute to a better understanding of regenerative mechanisms in starfish and the broader field of echinoderm genetics.

## Data description

### Experimental design and sample collection

In February 2021, live specimens of starfish, *A. amurensis* (~ 20 cm body height) were collected at Gijang of Busan, Korea, and maintained in a recirculating seawater system at 15℃ with ad libitum feeding until the experiment. The starfish were transferred to and kept in Plexiglas aquaria (30 × 20 × 20 cm) for 24 h before the start of the experiment. These were then divided into two groups: a control group (*n* = 3) and one arm amputated group (*n* = 3). After 3 days (72 h) by amputation, the nerve cord of starfish was collected from whole body and pooled for RNA-Seq analysis.

### RNA extraction, library construction, and sequencing

Total RNA was extracted using a Hybrid-R kit (GeneAll, Seoul, Korea) from A. amurensis following the manufacturer’s instructions. Total RNA integrity is checked Agilent 2100 Bioanalyzer (Agilent Technologies) with an RNA Integrity Number (RIN) ≥ 7. cDNA libraries were constructed using the TruSeq Stranded Total RNA LT Sample Prep Kit (Illumina, CA, USA) according to the manufacturer’s protocol. The paired-end 101 base reads were sequenced using the Illumina platform (Illumina). To obtain high-quality data, raw data was qualified using FASTQC (v0.11.7) and trimmed to remove adapter sequences and bases with base quality lower than three from the ends using the Trimmomatic program (v0.38): Using sliding window method, bases of reads that does not qualify for window size 4, and mean quality 15 are trimmed. The de novo assembly of clean reads was carried out using Trinity with default parameters without reference genome sequence. For assembled genes, clean reads are merged to non-redundant unique transcripts as long as possible and clustered into unigenes (i.e., unique genes) such that have a minimum length of 200 bp using CD-HIT-EST program (v4.6). Each unigenes corresponded to one or numerous transcript isomers (c*_g*_i*). ORF prediction for unigenes was performed using the TransDecoder program (v3.0.1) to identify candidate coding regions within the transcript sequence. Trimmed reads were aligned to the assembled reference using the Bowtie program (Trinity version trinityrnaseq_r20140717, bowtie 1.1.2) for subsequent analysis. For functional annotations, unigenes were searched against Gene Ontology (GO, v20180319), NCBI non-redundant Protein (NR, v20180503), UniProt (v20180116) and Kyoto Encyclopedia of Genes and Genomes (KEGG, v20200103) using BLASTN of NCBI BLAST (v2.9.0+) and BLASTX of DIAMOND (v0.9.21) software with an E-value default cutoff 1.0E-5.

### DEGs and functional enrichment analysis

For the differentially expressed gene analysis, the abundances of unigenes are estimated in the read count as an expression measure by the RNA-Seq by Expectation Maximization (RSEM) algorithm. The raw read count value of contigs obtained through the RSEM was used as the raw data. Statistical analysis is performed by Trinity (v2.15.1) with edgeR (dispersion value, 0.1) using q-value < 0.05 and |fold change (FC)|>2.

#### Real-time quantitative polymerase chain reaction (RT-qPCR)

To quantitatively analyze the expression of transcripts between two groups, RT-qPCR was employed using a QuantStudio™ 6 Flex Real-Time PCR system (Applied Biosystem, MA, USA). cDNA was synthesized by calculating 1 µg with the TOPscript™ cDNA synthesis Kit with oligo dT (dT18) (Enzynomics, Daejeon, Korea) according to the manufacturer’s instructions. The primer pairs are listed in Table 1. The RT-qPCR conditions were as follows with Prime Q-Master Mix (with SYBR Green I) (GenetBio, Daejeon, Korea): initial denaturation 95 ° C for 10 min; 45 cycles of 95 ° C for 15 s, 60 ° C for 30 s, and 72 ° C for 30 s. Triplicate amplifications were carried out independently, and the mRNA levels were normalized to actin, and the comparative threshold cycle method (2^−ΔΔCT^) determined the expression levels.

**Table 1:** Overview of data files/data sets

Label	Name of data file/data set	File types (file extension)	Data repository and identifier (DOI or accession number)
*Data file 1*	*Illumina sequencing reads for normal nerve cord*	Fastq file (fq.gz)	NCBI Sequence Read Archive (https://identifiers.org/insdc.sra:SRR29975745) [7]
*Data file 2*	*Illumina sequencing reads for arm amputated nerve cord*	Fastq file (fq.gz)	NCBI Sequence Read Archive (https://identifiers.org/insdc.sra:SRR29975744) [8]
*Data file 3*	Summary of A. amurensis transcriptomic analysis statistics	Document file (.docx)	Figshare (10.6084/m9.figshare.28390235) [9]
*Data file 4*	Statistics of the unigenes from assembled contigs by CD-HIT-EST and ORF prediction by transdecoder	Document file (.docx)	Figshare (10.6084/m9.figshare.28390235) [9]
*Data file 5*	Functional annotations of unigenes	Document file (.docx)	Figshare (10.6084/m9.figshare.28390235) [9]
*Data file 6*	*DEG analysis with Volcano*	Speadsheet (.xlsx)	Figshare (10.6084/m9.figshare.28390235) [9]
*Data file 7*	Primer list for RT-qPCR analysis	Document file (.docx)	Figshare (10.6084/m9.figshare.28390235) [9]
*Data file 8*	Validation of DEGs using qRT-PCR. Actin as a control for normalization.	Document file (.docx)	Figshare (10.6084/m9.figshare.28390235) [9]
*Data file 9*	De novo *transcriptome assembly of A. amurensis*	Fasta file (.fasta)	GenBank TSA (https://identifiers.org/ncbi/insdc:GLFZ00000000) [10]

### Gene expression analysis

In this study, *A. amurensis* de novo transcriptomic assembly was performed without a reference genome sequence (BioSample accession: SAMN42798991, BioProject ID: PRJNA1139949). RNA sequencing was conducted on two libraries using the Illumina platform, generating 84,440,518 and 75,924,806 raw reads, respectively. After quality filtering with Trimmomatic, 83,602,744 and 75,018,848 clean reads remained, with Q20 and Q30 base percentages exceeding 99% and 96%, respectively. The GC content was approximately 44% and 42% for the normal and post-amputated groups (Table 1, Data file 3). Clean reads were merged to construct a reference transcriptome, and de novo assembly using Trinity produced 359,822 transcript contigs. CD-HIT-EST was used to filter redundant sequences, resulting in 257,769 unigenes. The GC content of unigenes was 39.43%, the N50 value was 735 bp, and the average contig length was 570.79 bp. Open reading frame (ORF) prediction identified 17,628 ORFs using TransDecoder (Table 1, Data file 4). Functional annotation of unigenes was performed using BLASTX with DIAMOND for GO, UniProt, NR, and KEGG databases (Table 1, Data file 5). To investigate genes involved in starfish regeneration, DEGs were identified. Among the 257,769 unigenes, 147 contigs showed significant differential expression (|fc| ≥ 2, p(FDR) < 0.05) based on edgeR analysis (Table 1, Data file 6). Of these, 43 unigenes were upregulated, while 104 were downregulated in the normal group. To validate the RNA-seq results, five unigenes were selected for RT-qPCR, which confirmed consistency with the DEG analysis (Table 1, Data file 7–8). These results provide insights into the molecular mechanisms underlying regeneration in *A. amurensis*, contributing to the broader understanding of echinoderm biology [3, 5].

## Limitations

This study focused only on the transcriptome of the nerve cord in *A. amurensis*. However, the regeneration process involves interactions between multiple tissues, including muscles, epidermis, and immune cells. The lack of transcriptomic data from other tissues limits the comprehensive understanding of regeneration mechanisms. Also, since regeneration is a dynamic, time-dependent process, the absence of data from multiple time points (e.g., immediately after amputation, one week later, or one month later) restricts a complete understanding of the molecular events involved in regeneration.

## Data Availability

All sequencing reads are available in the NCBI Sequence Read Archive (SRA) under BioProject ID [PRJNA1139949] (https://identifiers.org/bioproject:PRJNA1139949). The transcriptome assembly and downstream analysis files are openly accessible via Figshare (10.6084/m9.figshare.28390235). The assembled transcriptome has been deposited in the GenBank Transcriptome Shotgun Assembly (TSA) database under accession number GLFZ00000000, which is part of the International Nucleotide Sequence Database Collaboration (INSDC).

## Abbreviations

NGS: Next-generation sequencing
DEG: Differentially expressed gene
ORF: Open reading frame
